# Exosomal microRNAs in colorectal cancer communication networks: implications for metastasis, therapy resistance, and precision medicine

**DOI:** 10.3389/fimmu.2026.1872130

**Published:** 2026-06-16

**Authors:** Ashutosh Kumar Maurya, Sheefa Mirza

**Affiliations:** 1 Department of Biochemistry and Molecular Biology, School of Biological Sciences, Central University of Kerala, Kerala, India; 2 Precision Biomarker Research Unit, Department of Internal Medicine, School of Clinical Medicine, Faculty of Health Sciences, University of the Witwatersrand, Johannesburg, South Africa

**Keywords:** microRNAs, extracellular vesicles, colorectal cancer, tumour microenvironment, liquid biopsy

## Abstract

Exosome-derived microRNAs (miRNAs) have recently been recognised as important players in the process of intercellular communication in CRC, thus allowing tumour cells to affect not only each other but also the whole milieu of interactions occurring in the tumour microenvironment and metastatic niches. The growing body of evidence points to selective packaging of exosomal miRNAs via controlled biogenesis mechanisms and modulation by oncogenic signalling and microenvironmental stress. After being delivered to target cells, these miRNAs affect interrelated signalling pathways involved in the epithelial-mesenchymal transition, stemness, immune escape, angiogenesis, metastasis niche development, and drug resistance. Instead of being regarded as simple biomolecular markers, exosomal miRNAs are better understood as regulators of networks of processes that allow tumour cell populations to coordinate their response to external stimuli and therapeutic interventions. This view can shed light on tumour heterogeneity, metastasis, and resistance to treatment in CRC. At the same time, their high biological stability in blood plasma has raised hopes for their clinical utility as markers of liquid biopsy and therapeutic targets. However, further progress in clinical translation is hampered by several obstacles, such as extracellular vesicle heterogeneity, methodological variability, and lack of standardisation. This review synthesises current knowledge on exosome biogenesis, selective miRNA sorting, tumour microenvironment communication, and therapy resistance in CRC. In addition, it highlights emerging systems biology, single-vesicle, and artificial intelligence–based approaches that may improve biomarker robustness and translational relevance. Collectively, this review argues that integrative and mechanism-driven strategies will be necessary to advance exosomal miRNAs from exploratory biomarkers toward clinically meaningful applications in precision oncology.

## 1 Introduction: exosomal miRNAs as dynamic regulators of CRC biology

CRC is one of the leading causes of cancer-related mortality worldwide and represents a major global health burden (1). It is a biologically heterogeneous disease driven not only by intrinsic genetic and epigenetic alterations within tumour cells but also by complex interactions with the surrounding tumour microenvironment (2). Although genomic and transcriptomic studies have shed light on the development and progression of CRC, they are based on static features of individual cells and have limited ability to reveal dynamic intercellular communication processes, which play an important role in tumour development, metastasis, and treatment response (3, 4).

EVs, such as exosomes, have been shown to play an important role in cell-to-cell communication during cancer development. Exosomes are small membrane-bound vesicles that range from 30 to 150 nm in diameter, which are formed from the endosomal compartment and secreted into the extracellular environment. Secreted exosomes can be taken up by other cells, locally or distally, to affect the behaviour of the recipient cells (5, 6). Molecular analysis of the full complement of exosomal molecules has revealed that they contain a unique profile of proteins, lipids, and nucleic acids that reflect the donor cells’ origin and physiological status (7, 8). The miRNA component of exosomal molecular content has been identified as important, especially given its established role in gene regulation (9). Importantly, recent evidence has shown that the miRNA content of exosomes is not randomly distributed but rather contains a selective population of cellular miRNAs, suggesting the existence of a regulatory process in miRNA sorting into exosomes (10). This selective packaging implies that exosomal miRNAs are not merely cellular waste products but may function as active signalling molecules that can alter gene expression programs in recipient cells (11).

In CRC, numerous studies (primarily preclinical) have shown that tumour-derived exosomal miRNAs participate in key biological processes associated with disease progression. These include promotion of epithelial-to-mesenchymal transition (EMT), modulation of the tumour microenvironment (TME), stimulation of angiogenesis, suppression of anti-tumour immune responses, and establishment of pre-metastatic niches, particularly within the liver (12, 13). Experimental models using co-culture systems and *in vivo* models (in CRC and other cancer systems) have shown that exosomal miRNAs can be taken up by different cells in the tumour microenvironment, leading to transcriptional and phenotypic reprogramming of recipient cells. In addition to their involvement in TME interactions, recent studies (including evidence from CRC models, with additional support from other cancer systems) have shown that exosomal miRNAs play a role in maintaining stemness and phenotypic plasticity in CRC cells. These vesicles may therefore contribute to tumour heterogeneity and therapeutic resistance, thereby promoting CRC persistence (14, 15).

The stability of exosomal miRNAs in the circulation has led to their recognition as promising non-invasive liquid biopsy biomarkers for CRC diagnosis and prognosis. These molecules are resistant to enzymatic degradation due to their encapsulation in lipid vesicles and can therefore be detected in the circulation and other body fluids (16, 17). Previous clinical studies have demonstrated the association of exosomal miRNAs in the bloodstream with the diagnosis, prognosis, recurrence, and treatment of CRC (18). Despite these promising results, only a few potential biomarkers have been shown to have reproducibility in different studies (19). Recent studies indicate that several factors may contribute to translational challenges, including the biological heterogeneity of extracellular vesicle populations, methodological heterogeneity in isolation and quantification approaches, and a lack of mechanistic validation of candidate miRNAs regarding their involvement in cancer pathogenesis (20, 21). Moreover, most studies on miRNAs have concentrated on individual miRNAs, which have overlooked their context-dependent role in regulating tumour ecosystems, possibly simplifying the complex process of exosome-mediated cell-to-cell communication in cancer cells (22).

The above findings highlight a critical gap in recognising exosomal miRNAs not only as passive molecular biomarkers, but as active regulators of the tumour ecosystem and its behaviour. Despite growing evidence of their importance, there remains a limited integrated understanding of their biogenesis, selective loading, and functional transfer, which constrains their biological interpretation and clinical translation in colorectal cancer (CRC).

In this review, we aim to address these gaps by synthesising current knowledge on: (i) the molecular mechanisms governing exosome biogenesis and miRNA selection, (ii) the role of exosomal miRNAs in tumour microenvironment (TME) communication and therapy resistance, and (iii) their clinical applicability as diagnostic, prognostic, and therapeutic tools. Furthermore, we highlight existing challenges and discuss emerging systems-based approaches that may facilitate the translation of these mechanisms into clinically relevant, precision medicine frameworks.

## 2 Molecular mechanisms of exosome biogenesis and miRNA sorting in CRC

### 2.1 Exosome biogenesis pathways and regulatory machinery

**Figure 1:**
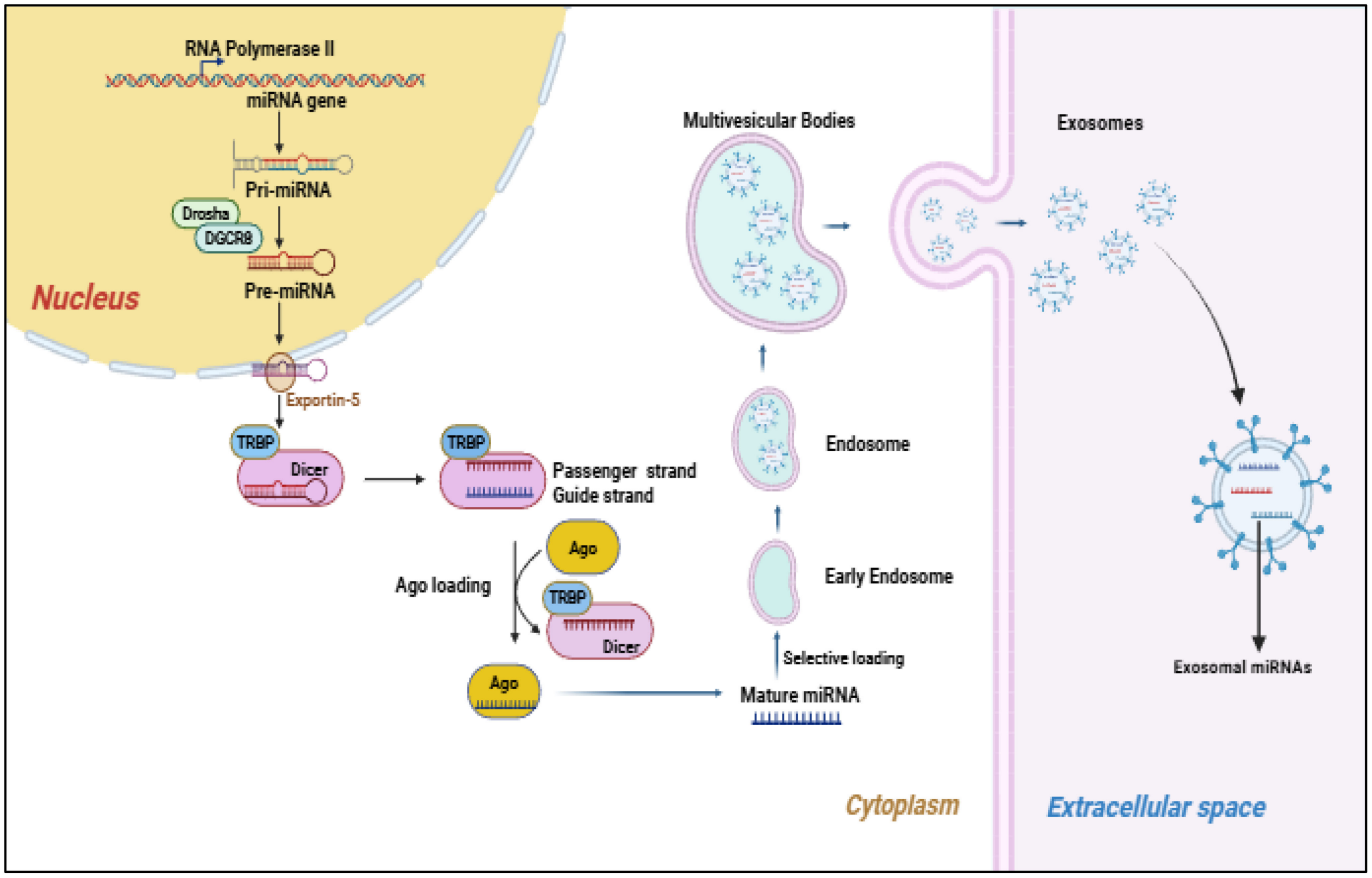
Biogenesis of miRNAs and their incorporation into exosomes: The biogenesis of miRNAs occurs in the nucleus, where RNA polymerase II transcribes miRNA genes into primary miRNA transcripts, known as pri-miRNA. This pri-miRNA is cleaved by the Drosha/DGCR8 complex into a precursor miRNA, known as a pre-miRNA. This pre-miRNA is transported into the cytoplasm by Exportin-5. Once transported into the cytoplasm, the pre-miRNA is further cleaved into a miRNA duplex, consisting of a guide and passenger strand, by the action of Dicer. The guide strand is preferentially incorporated into the Argonaute protein, forming the RISC complex that gives rise to the mature miRNA. Concurrently, endosome maturation occurs, forming early endosomes and MVBs that contain intraluminal vesicles. Selectively packed miRNAs are incorporated into these vesicles, which are then secreted into the extracellular environment as exosomes after fusion of MVBs with the plasma membrane.

Exosomes are small EVs, originating from the endosomal system. They are generated through inward budding of the endosomal membrane during the maturation of early endosomes into multivesicular bodies (MVBs), which contain intraluminal vesicles. Upon fusion of MVBs with the plasma membrane, these vesicles are released into the extracellular space as exosomes (23) (**Figure 1**). This endosomal origin distinguishes exosomes from other EVs, such as microvesicles, which are shed directly from the plasma membrane (24).

Exosome formation is tightly regulated by several molecular pathways, most prominently the endosomal sorting complex required for transport (ESCRT) machinery. The ESCRT machinery is comprised of four main complexes: ESCRT-0, ESCRT-I, ESCRT-II, and ESCRT-III, as well as accessory proteins that are involved in coordinating vesicle scission and membrane deformation/cargo recognition (25). Disruption of ESCRT machinery function affects exosome secretion and composition, underscoring its importance in vesicle biogenesis (26). Apart from ESCRT-dependent pathways, ESCRT-independent pathways also contribute to exosome biogenesis. These include tetraspanins such as CD9, CD63, and CD81, as well as lipid-based mechanisms such as ceramide generation (27, 28). The existence of multiple exosome biogenesis pathways in experimental systems suggests that exosome formation is a flexible, context-dependent process that responds to signalling requirements and cellular states (29).

### 2.2 Oncogenic and microenvironmental regulation of exosome secretion in CRC

In CRC, the process of exosome biogenesis and release is often altered compared with that in normal intestinal epithelial cells. The oncogenic signalling pathways activated in CRC, such as KRAS, Wnt/β-catenin, and p53, have been shown to affect vesicle trafficking and secretion (30, 31). For example, mutant KRAS has been associated with increased exosome release and altered exosomal cargo composition, thereby influencing communication between tumour cells and their surrounding microenvironment (32, 33).

The tumour microenvironment also influences exosome secretion patterns. Hypoxia, a condition often found in solid tumours, has been shown to increase exosome secretion and alter the RNA content using hypoxia-inducible factors (34). Exposures to chemotherapeutic agents also increase exosome secretion, leading to adaptive intercellular communication in the tumour (35). This suggests that the secretion of exosomes in CRC is not a passive event but is regulated by both oncogenic and external signals in a dynamic manner.

### 2.3 Selective miRNA sorting and context-dependent packaging mechanisms

It has been found that miRNAs are not randomly packaged into exosomes but are selectively packaged. This has been supported by comparative profiling studies, which have found that the miRNA content of exosomes is different from that of their parent cell type, both in normal and cancer states (36, 37). This selective enrichment supports the existence of active sorting mechanisms. Several RNA-binding proteins are responsible for the selective loading of miRNAs into exosomes. Heterogeneous nuclear ribonucleoproteins, especially hnRNPA2B1, recognise specific sequence motifs in miRNAs and assist in their loading into exosomes via post-translational modifications such as sumoylation (38, 39). Another RNA-binding protein, Y-box binding protein 1 (YBX1), is also involved in the sorting of exosomal miRNAs. YBX1 depletion decreases specific miRNAs in exosomes (40).

Further research indicates that Argonaute proteins and the RNA-induced silencing complex play a role in miRNA sorting, although their functions are unclear and context-dependent (41). These findings support the hypothesis that miRNA packaging into exosomes is a controlled, selective process. The composition of exosomal miRNAs is also dependent on the physiological and pathological status of the donor cells. Stress conditions in cancer cells, including hypoxia, inflammation, and DNA damage, have been reported to influence exosomal miRNA composition and secretion in tumour models, including CRC, reflecting regulated and adaptive sorting mechanisms under pathological conditions (42, 43). This suggests that the selective export of miRNAs may represent an adaptive mechanism.

In CRC models, treatment-induced stress has been associated in CRC models and other cancer systems with enhanced secretion of exosomal miRNAs linked to survival, epithelial-mesenchymal transition, and drug resistance (44, 45). Such selective release may reduce intracellular levels of tumour-suppressive miRNAs while simultaneously modulating neighbouring cells within the tumour microenvironment. Despite these advances, several key issues remain to be clarified. For instance, how is specificity in cytoplasmic miRNAs determined, how is this process coordinated with vesicle biogenesis, and do different subpopulations of exosomes have distinct miRNA content (46)? Much of the mechanistic insight derives from *in vitro* models, and its *in vivo* relevance in human CRC remains to be further validated for interpreting exosomal miRNA profiles in translational contexts.

## 3 Exosomal miRNAs in tumour ecosystem communication

### 3.1 Tumour-tumour communication and stemness propagation

Communication between CRC cells is mediated through exosomal miRNA. CRC cells release exosomes, which are taken up by other malignant cells, leading to alterations in gene expression and cell behaviour (47). Horizontal transfer is a mechanism for coordinating adaptive responses in tumour cell populations to environmental and therapeutic stress.

Multiple studies demonstrate that exosomal miRNA transfer contributes to the regulation of EMT in CRC. Moreover, exosomal miRNAs derived from aggressive tumour cell subclones have been found to target and downregulate epithelial markers and upregulate mesenchymal genes, thus promoting cell migration and invasion (48, 49). Notably, these effects occur even in the absence of cell-cell contact, highlighting the role of exosome-based cell communication in cancer progression. Besides EMT, exosomal miRNAs play a role in maintaining tumour heterogeneity. The exchange of miRNAs regulating gene expression between different subpopulations of cancer cells allows more aggressive subpopulations to influence less aggressive subpopulations, thereby creating diversity among cells of the same tumour (50). This collective adaptation supports a population-level model of tumour evolution.

Emerging evidence further indicates that exosomal miRNAs are involved in maintaining stem-like properties in CRC cells. Cancer stem-like cells exhibit enhanced self-renewal observed in CRC and other cancer models, tumour-initiating capacity, phenotypic plasticity, and resistance to therapy. The process of exosome-mediated miRNA delivery further supports this by targeting key signalling pathways involved in stemness and survival. For instance, CRC-derived exosomes carrying miR-19b increase stemness and radioresistance by targeting FBXW7 and activating the Wnt/β-catenin pathway in target cells (51). Similarly, mesenchymal stem cell-derived exosomes containing miR-486-5p, reported in CRC experimental system, inhibit CRC stemness and glycolysis by targeting NEK2, demonstrating the context-dependent nature of exosomal miRNA effects (52). In addition, tumour stem cell-derived exosomal miR-17-5p promotes tumour growth and modulates immune responses in CRC (53). This is in line with other research showing that exosomal miRNAs modulate CSCs in several ways, including through the Notch, TGFβ, and PI3K/AKT signalling pathways (54).

These observations, in total, suggest that exosomal miRNAs are important in coordinating cell phenotypes in tumours and in propagating stemness traits that drive drug resistance and tumour persistence.

### 3.2 TME crosstalk: fibroblasts, endothelium, and immune cells

There is extensive communication between CRC cells and the components of the tumour microenvironment through exosomal miRNAs. Cancer-associated fibroblasts, endothelial cells, and immune cells can take up tumour-derived exosomes observed in CRC models, thus causing functional reprogramming of the cells that take up the exosomes (55). Tumour-derived exosomal miRNAs induce activation of fibroblasts into cancer-associated fibroblasts, which secrete extracellular matrix components and soluble factors that support tumour invasion and growth (56). Conversely, fibroblast-derived exosomes carrying miRNAs can reinforce malignant traits in CRC cells, including enhanced invasion and therapy resistance (57). This two-way communication creates a self-supporting environment for tumours. Another important target is endothelial cells. Endothelial cells take up CRC-derived exosomes, which also alter angiogenic signalling pathways, thereby driving vascular remodelling and neovascularisation (58). These changes enable tumour growth and metastasis.

Another important role of exosomal miRNAs in CRC involves immune modulation within the tumour microenvironment. Tumour-derived exosomes have been shown to regulate the differentiation, activation, and functional behaviour of multiple immune cell populations, thereby contributing to immune evasion and tumour progression (59, 60). Macrophages exposed to CRC-derived exosomes frequently acquire immunosuppressive M2-like phenotypes characterised by altered cytokine secretion, enhanced tissue remodelling, and suppression of anti-tumour immune responses (61). Exosomal miRNAs contribute to macrophage polarisation through modulation of signalling pathways such as STAT3, PI3K/AKT, and NF-κB, which collectively support tumour-promoting inflammatory environments (59, 61).

In addition to macrophages, tumour-derived exosomal miRNAs can impair dendritic-cell maturation and antigen-presenting capacity, thereby limiting effective T-cell priming and adaptive immune activation (60, 62). Emerging evidence also suggests that exosomal miRNAs suppress cytotoxic CD8+ T-cell responses either indirectly through modulation of antigen-presenting cells or directly through inhibitory signalling pathways that promote T-cell dysfunction and exhaustion (62). Furthermore, exosomal signalling has been implicated in reduced natural killer (NK) cell cytotoxicity and altered immune surveillance mechanisms within the CRC microenvironment (60, 62). Several studies additionally indicate that tumour-derived exosomes contribute to expansion of regulatory T cells and myeloid-derived suppressor cells, both of which reinforce immunosuppressive conditions favourable for tumour growth (62).

**Figure 2:**
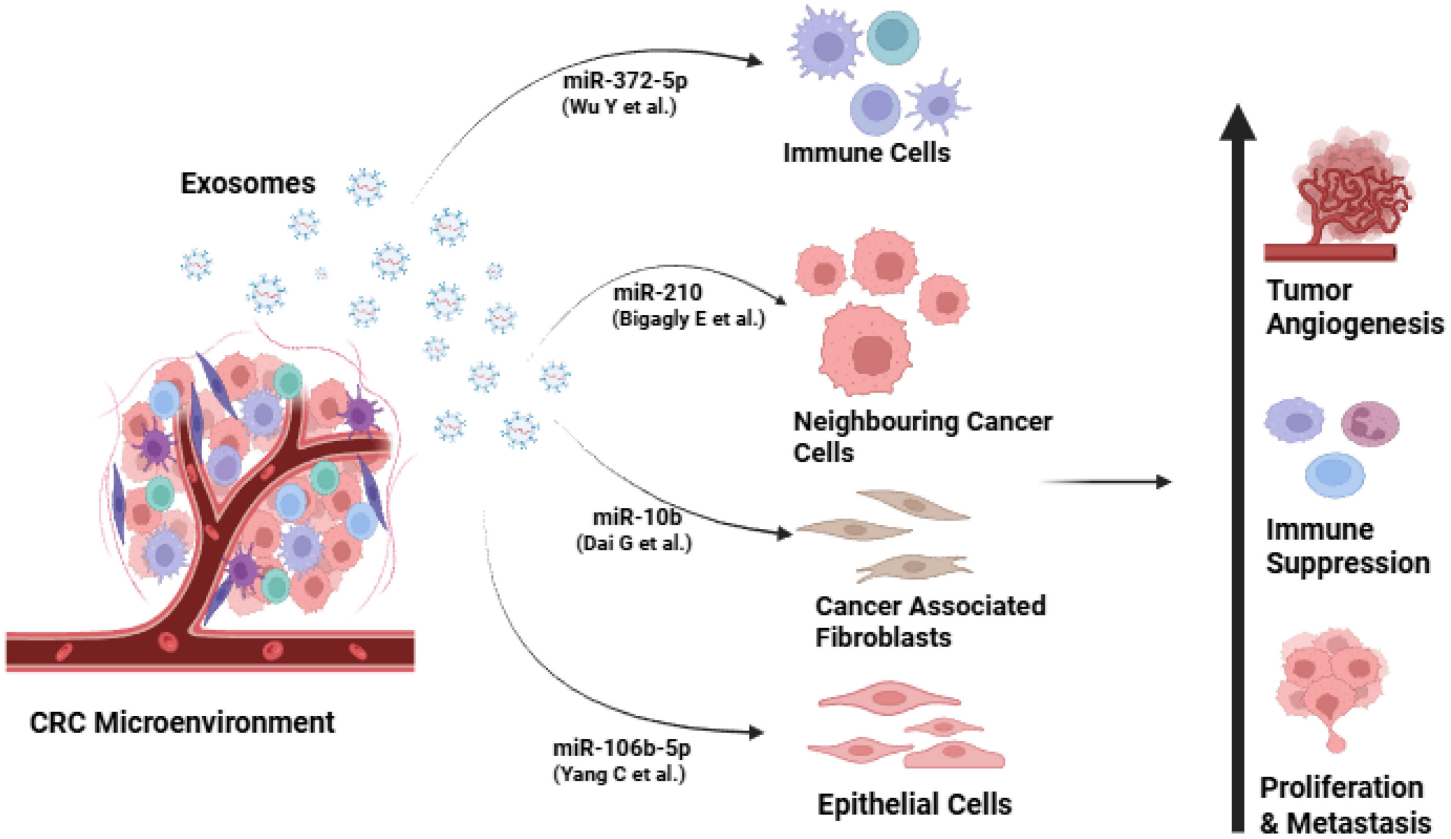
Tumour-derived exosomal miRNAs regulate cell reprogramming in the CRC microenvironment: Exosomes released by CRC cells selectively accumulate certain miRNAs, which regulate various components of the tumour microenvironment. Exosomal miRNAs, in turn, regulate various cell types in the tumour microenvironment: miR-372-5p regulates immune cells, which contribute to immune suppression; miR-210 regulates adjacent cancer cells, which adapt to their microenvironment; miR-10b regulates cancer-associated fibroblasts; and miR-106b-5p regulates epithelial cells, which strengthen malignant characteristics. The cooperative action of tumour-derived exosomal miRNAs controls various aspects of the tumour microenvironment, including tumour-induced angiogenesis, immunosuppression, cell proliferation, and metastasis. The multi-directional cell signalling network clearly indicates the significance of exosomal miRNAs in regulating the CRC ecosystem.

Importantly, exosomal miRNAs have also been linked to immune checkpoint regulation. Tumour-associated exosomal signalling may enhance expression of checkpoint molecules such as PD-L1 and contribute to suppression of anti-tumour immunity through context-dependent inflammatory signalling networks (62). Through these coordinated interactions, exosomal miRNAs contribute not only to angiogenesis and stromal remodelling but also to the establishment of an immunosuppressive tumour ecosystem that facilitates tumour persistence, metastatic dissemination, and therapeutic resistance (**Figure 2**) (63–66).

### 3.3 Pre-metastatic niche formation and context-specific signalling

CRC has an inherent predilection for metastatic spread to the liver, and CRC-derived exosomes have been reported to facilitate pre-metastatic niche formation in target organs for metastatic colonisation (67, 68). Experimental studies demonstrate that CRC-derived exosomes preferentially accumulate in the liver, where they are internalised by resident stromal and immune cells (69). The miRNAs delivered through these vesicles modulate inflammatory pathways, extracellular matrix remodelling, and immune surveillance mechanisms, thereby creating a microenvironment permissive for metastatic seeding and growth (70). These findings support a role in CRC-derived experimental systems for exosomal miRNAs in establishing pre-metastatic niches before the arrival of disseminated tumour cells.

Despite increasing evidence of functional relevance, several issues remain regarding the specificity and directionality of exosomal miRNA signalling. Not all exosomal miRNA is functionally relevant, and vesicle uptake is not necessarily followed by significant transcriptional reprogramming of the recipient cells (71). This may be dependent on exosome concentration, uptake efficiency, and cell status and internal processing abilities. Moreover, different miRNAs can have different effects observed in CRC and other tumour models depending on the cellular context and microenvironmental cues (72, 73). These considerations highlight the necessity of functional validation in addition to profiling studies when interpreting exosomal miRNA-mediated communication.

Altogether, current evidence supports a model in which exosomal miRNAs act as key mediators of tumour ecosystem communication. By coordinating tumour cell populations, reprogramming stromal and immune compartments, and preparing distant organs for metastasis, exosomal miRNAs operate as systemic regulators of CRC progression. Key exosomal miRNAs implicated in TME communication and therapy resistance in CRC are summarised in **Table 1** and the exosomal microRNAs implicated in immune regulation are depicted in **Table 2**.

**Table 1:** Representative exosomal miRNAs in CRC progression, cell type of origin, target cell type, molecular pathways, and functional roles in CRC progression.

Exosomal miRNA		Donor cell type	Recipient cell type	Target gene(s)/pathway	Functional outcome	Reference
miR-19b	CRC cells		CRC cells	FBXW7; Wnt/β-catenin signalling	Enhanced stemness; radioresistance	(51)
miR-486-5p	Mesenchymal stem cells		CRC cells	NEK2; glycolysis-associated pathways	Reduced stemness; inhibition of glycolysis	(52)
miR-17-5p	Tumour stem-like cells		Immune cells/CRC cells	Immune-modulatory pathways	Promotion of tumour growth; suppression of anti-tumour immunity	(53)
miR-372-5p	CRC cells		Immune cells	Immune signalling pathways	Immune suppression	(63)
miR-210	CRC cells		Neighbouring cancer cells	Hypoxia-associated signalling	Tumour adaptation; survival	(64)
miR-10b	CRC cells		Cancer-associated fibroblasts	EMT-related signalling	Fibroblast reprogramming; tumour support	(65)
miR-106b-5p	CRC cells		Epithelial cells	Proliferation-associated signalling	Enhanced proliferation and malignant phenotype	(66)

The selected miRNAs demonstrate how exosomes secreted by CRC cells can modulate cell-to-cell communication in the tumour microenvironment, thereby promoting stemness, immunomodulation, fibroblast reprogramming, metabolic rewiring, and drug resistance.

**Table 2:** Summarises representative exosomal miRNAs implicated in immune regulation within the CRC tumour microenvironment, including donor and recipient cell types, affected pathways, immune consequences, experimental context, and current level of evidence.

Exosomal miRNA	Exosome donor cell	Recipient immune cell	Target gene/pathway	Immune function affected	Experimental model/context mentioned in text	Level of evidence
miR-17-5p	Tumour stem-like cells	Immune cells	Immune-modulatory pathways	Promotion of tumour growth; suppression of anti-tumour immunity	CRC experimental studies described in tumour stem cell-derived exosome models	Preclinical mechanistic evidence
miR-372-5p	CRC cells	Immune cells	Immune signalling pathways	Immune suppression	Tumour microenvironment communication studies in CRC	Preclinical functional evidence
not individually specified	CRC cells	Macrophages	Cytokine secretion and antigen presentation pathways	Induction of immunosuppressive macrophage phenotypes; facilitation of immune evasion	Exposure of macrophages to CRC-derived exosomes	Preclinical mechanistic evidence
not individually specified	CRC cells	Dendritic cells	Immune regulatory signalling pathways	Modulation of dendritic cell differentiation and function	Tumour exosome-mediated immune modulation studies	Preclinical functional evidence
not individually specified	CRC cells	Cytotoxic T lymphocytes	T-cell signalling pathways	Impairment of cytotoxic T-cell responses	Direct and indirect modulation through tumour-derived exosomes	Preclinical mechanistic evidence
not individually specified	CRC cells	Fibroblasts and macrophages	Protective and immunosuppressive signalling pathways	Promotion of therapy-protective microenvironment during chemotherapy	Therapy resistance-associated exosome signalling studies	Preclinical translational evidence
not individually specified	CRC cells	Immune and stromal cells in liver microenvironment	Inflammatory pathways and immune surveillance mechanisms	Formation of immunosuppressive pre-metastatic niche	Experimental liver metastasis and pre-metastatic niche models	Preclinical *in vivo* evidence

## 4 Exosomal miRNAs in tumour progression and therapy resistance

### 4.1 Regulation of EMT, invasion, and metastatic dissemination

Tumour progression in CRC is characterised by dynamic changes in intercellular communication that promote invasive and metastatic phenotypes. Increasing evidence indicates that CRC-derived exosomal miRNAs actively contribute to these processes by reprogramming both tumour and stromal compartments (74). Experimental models have shown that following exposure to exosomes derived from CRC, alterations in gene expression and target cell behaviour can be detected (75). Exosomal miRNAs play a vital role in regulating EMT, a key event in cancer cell dissemination. Studies show that tumour-derived exosomal miRNAs suppress epithelial markers such as E-cadherin while enhancing mesenchymal marker expression in recipient CRC cells, thereby increasing migratory and invasive capacity (76). These findings support the concept that exosomal miRNAs facilitate the acquisition of aggressive phenotypes within tumour cell populations.

In addition to EMT, exosomal miRNAs regulate critical oncogenic signalling pathways that support tumour development and survival. CRC-derived exosomes carrying oncogenic miRNAs induce target cell signalling cascades that include PI3K/AKT, MAPK, and Wnt/β-catenin pathways, which support tumour cell proliferation and evasion of apoptosis (77). Most interestingly, this mode of regulation can overcome differences in genetic backgrounds between donor and target cells, suggesting that it can override intrinsic regulatory mechanisms. Metastasis remains the principal cause of mortality in CRC, with the liver representing the most common site of distant spread. Accumulating evidence indicates that CRC-derived exosomal miRNAs participate in multiple stages of the metastatic cascade, including local invasion, intravasation, survival in circulation, and colonisation of distant organs (78).

Preclinical studies show that tumour-derived exosomes carrying specific miRNAs increase vascular permeability, thereby facilitating tumour cell extravasation at secondary sites (79). Within the liver microenvironment, exosomal miRNAs delivered to stromal cells induce inflammatory and fibrotic responses that promote the formation of a pre-metastatic niche (80). These findings suggest that exosomal miRNAs contribute to metastatic progression by influencing both tumour-intrinsic properties and host tissue responses. Clinical observations further support these experimental findings. Specifically, differences in circulating levels of exosomal miRNAs have been linked to metastatic burden and disease stage in CRC patients (81). Even though these findings are largely correlative, they highlight the biological importance of exosomal miRNA signalling in advanced disease.

**Figure 3:**
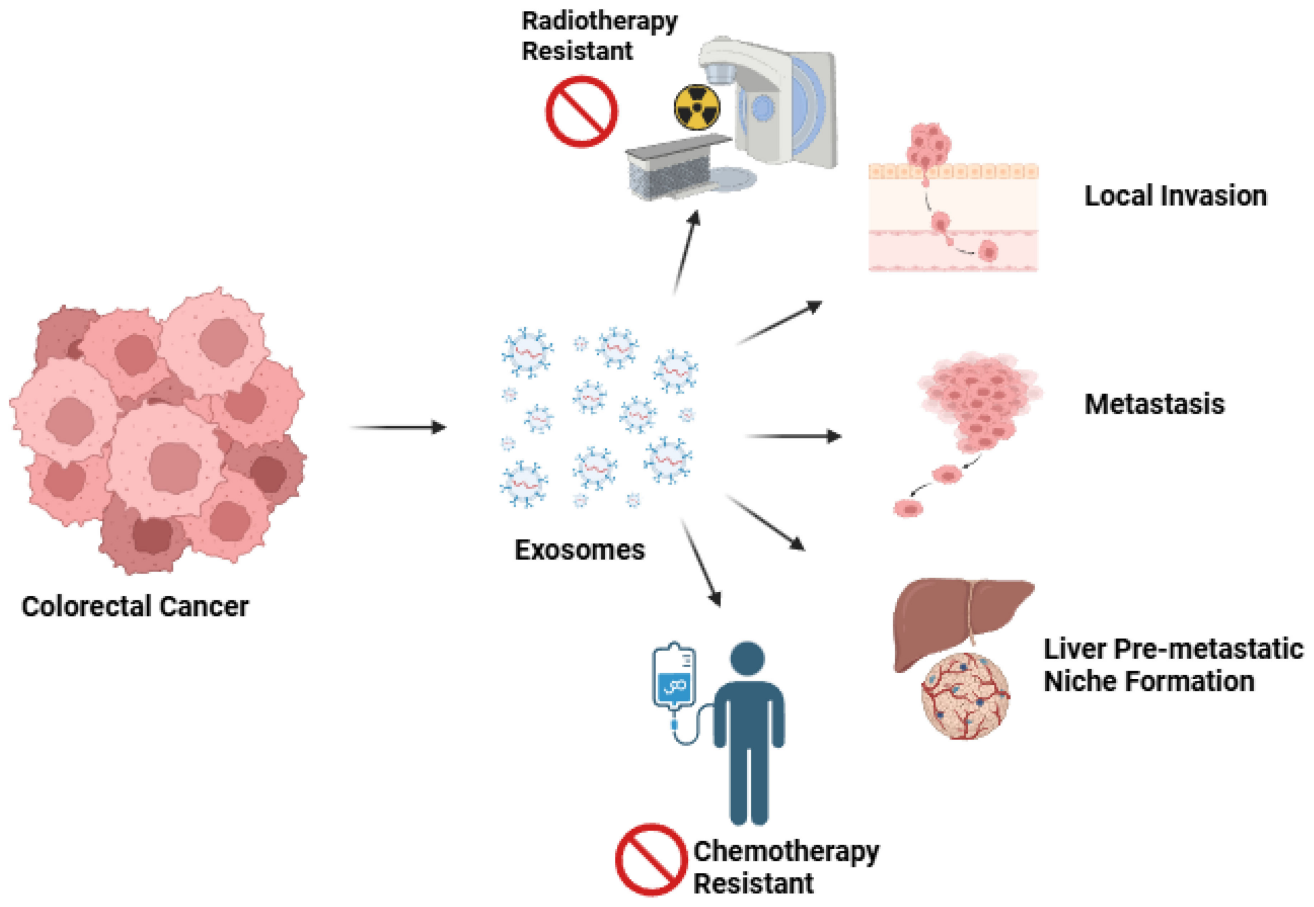
Functional consequences of tumour-derived exosomal miRNAs on the progression of CRC and therapeutic resistance: The cells of CRC actively secrete exosomes, which are enriched with selectively secreted miRNAs, into the microenvironment of the tumour and the systemic circulation. Exosomal miRNAs in CRC play a crucial role in the development of various hallmarks of disease progression. The exosomal miRNAs of CRC play a significant role in local invasion, facilitating epithelial-mesenchymal transition and thereby enhancing the tumour cells’ invasive properties. The exosomal miRNAs of CRC also play a crucial role in the systemic dissemination of the tumour cells, which is associated with the formation of pre-metastatic niches, especially in the liver. Moreover, exosomal miRNAs in CRC facilitate the horizontal transmission of resistance-associated regulatory signals, thereby conferring resistance to chemotherapy and radiotherapy.

Overall, tumour-derived exosomal miRNAs regulate local invasion, metastasis, pre-metastatic niche formation, and the horizontal transfer of resistance to therapy, which together drive tumour adaptation at the population level (**Figure 3**).

### 4.2 Exosomal miRNAs in resistance to systemic therapy and radiotherapy

Resistance to systemic therapy represents a major challenge in CRC management. Resistance-associated exosomal miRNA signalling has been investigated across multiple clinically relevant CRC treatment settings, including fluoropyrimidine-based chemotherapy, platinum-based therapy, irinotecan-containing regimens, targeted biological therapies, and radiotherapy. Although the mechanistic evidence remains predominantly preclinical, CRC-specific studies, together with supportive evidence from other cancer types, collectively suggest a role for exosomal miRNAs in adaptive tumour responses to therapeutic stress. Exosomal microRNAs are involved in both intrinsic (tumour-related) and acquired (treatment-induced) chemotherapy resistance. Most available evidence has focused on resistance to fluoropyrimidine-based therapy (5-fluorouracil), oxaliplatin-containing regimens, and irinotecan-based chemotherapy (82). Drug-resistant CRC cell lines release exosomes that have an array of microRNAs targeting genes involved in apoptosis, DNA damage response, and drug transport, as demonstrated in colorectal cancer experimental models. These exosomes can reduce the sensitivity of drug-sensitive cancer cells when transferred. These findings support the concept that cancer cell resistance traits can be transferred to other drug-sensitive cancer cells via the horizontal transfer of exosomal microRNA (83), a mechanism that has also been reported in other cancer types, including breast cancer models, but requires further CRC-specific validation. Exosomal miRNAs also modulate therapeutic responses indirectly by altering the tumour microenvironment, including in CRC experimental models. The delivery of specific miRNAs to fibroblasts and macrophages has been shown to confer protective and immunosuppressive phenotypes that protect tumour cells from chemotherapy-induced stress (84), although some of these findings derive from non-CRC cancer models and should be interpreted as supportive evidence. This indicates the intricate relationship between tumour cells and the ecosystem in determining the response to therapy (85).

Beyond cytotoxic chemotherapy, emerging evidence suggests that exosomal miRNAs may also influence responsiveness to targeted therapies through modulation of oncogenic signalling pathways, tumour microenvironment interactions, and adaptive survival programs. However, evidence specifically addressing resistance to EGFR-targeted therapies, VEGF-targeted therapies, and immune checkpoint blockade in CRC remains comparatively limited and requires further mechanistic and clinical validation, as most current mechanistic insights are extrapolated from broader oncological studies.

Neoadjuvant chemoradiotherapy represents a major treatment modality in locally advanced rectal cancer, where radioresistance remains a clinically significant challenge. New findings show that exosomal miRNAs play a role in the response to radiation by regulating DNA repair and oxidative stress mechanisms (86), including in CRC experimental models. Preclinical studies have shown that irradiated CRC cells release exosomes with specific miRNA profiles that enhance DNA repair capacity in target cells (87). The transfer of radioresistance-associated miRNAs via exosomes has been linked to radiotherapy failure and cancer recurrence (88, 89), although some supporting evidence originates from non-CRC cancer models and should be interpreted cautiously. Though the clinical significance of these findings is not well established, they support the view that exosomal miRNAs play a general role in the response to stress across cancer cell populations, including but not limited to CRC.

### 4.3 Conceptual framework: population-level tumour adaptation

Collectively, these findings support a model in which exosomal miRNAs function as mediators of tumour adaptation at the population level rather than as isolated molecular factors. By horizontally disseminating aggressive and treatment-resistant traits, exosomal miRNAs enable CRC cell populations to collectively respond to environmental and therapeutic pressures. This model challenges the traditional view of resistance as a cell-autonomous event and underscores the role of intercellular communication networks in cancer progression. In relation to its application, exosome-mediated communication may represent a target for intervention against shared resistance mechanisms and cancer cells’ adaptive capabilities (90).

## 5 Clinical translation: exosomal miRNAs as liquid biopsy biomarkers in CRC

### 5.1 Biological rationale and diagnostic potential

The detection of CRC at early stages and the constant monitoring of the disease remain a challenge, particularly because of the invasive nature of the biopsy of tumour tissues and the low sensitivity of the available serum markers, such as carcinoembryonic antigen (91, 92). Exosomal miRNAs have emerged as attractive biomarker candidates due to their stability in circulation, resistance to RNase-mediated degradation, and relative enrichment compared with freely circulating miRNAs (93). Indeed, experimental evidence suggests that encapsulation of miRNAs within exosomal lipid bilayers makes them resistant to degradation under harsh conditions, such as freeze-thaw cycles and long-term storage (94). Tumour-derived exosomes are also found in various biofluids, such as plasma, serum, and stool, enabling non-invasive sampling of CRC patients (95). These properties collectively provide a strong biological rationale for evaluating exosomal miRNAs as diagnostic biomarkers.

High-throughput profiling approaches, including next-generation sequencing and quantitative PCR, have identified circulating exosomal miRNA signatures capable of distinguishing CRC patients from healthy individuals with moderate to high sensitivity and specificity (96). Interestingly, some studies indicate that exosomal miRNA panels can be used to identify individuals with early-stage CRC or adenomas in asymptomatic individuals (97). In addition, stool-derived exosomal miRNA has been proposed as a supplementary diagnostic tool to Faecal Occult Blood Tests. However, validation in larger cohorts remains limited (98). Despite promising results from various studies, there is considerable variation in miRNA expression profiles, which can be attributed to heterogeneity across population studies, sample handling procedures, detection platforms, and normalisation strategies (99). This variability limits reproducibility and hinders clinical translation.

### 5.2 Prognostic and predictive applications

Apart from their role in CRC diagnosis, exosomal miRNAs have been evaluated for their prognostic and predictive potential in CRC patients. Some studies have shown an association between circulating exosomal miRNA levels and CRC stage, lymph nodes, metastasis, and survival in CRC patients (100). These associations suggest that exosomal miRNA profiles may reflect tumour aggressiveness and systemic disease activity (101). Exosomal miRNAs have also been explored as predictors of therapeutic response. Alterations in circulating exosomal miRNA levels before or during treatment have been linked to resistance to chemotherapy and radiotherapy in CRC (102). Longitudinal analyses indicate that changes in exosomal miRNA profiles may precede radiological or clinical evidence of treatment failure, highlighting their potential utility in real-time disease monitoring (103).

Nevertheless, the majority of prognostic and predictive studies have been retrospective in design with relatively small numbers of patients. In addition, there have been few studies with adequate multivariate analysis that have controlled for established clinicopathological variables (104). Consequently, although the associations are compelling, validation studies are needed.

### 5.3 Comparison with other liquid biopsy modalities

**Figure 4:**
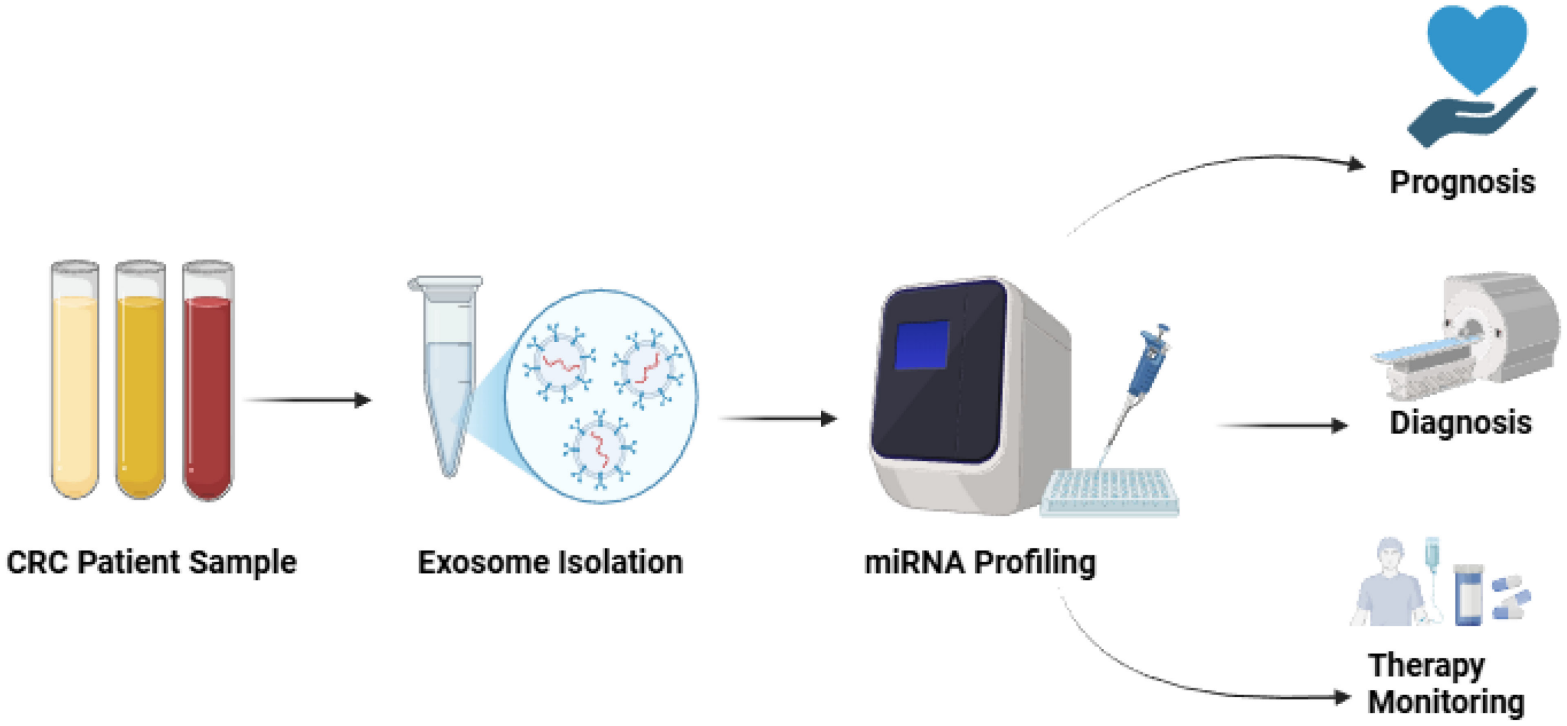
Clinical workflow and translational applications of exosomal miRNAs in CRC. Exosomal miRNAs can be isolated from biofluids collected from CRC patients. These biofluids include plasma or serum. After exosome isolation, exosomal miRNA profiling can be performed using quantitative PCR or other analytical methods. The exosomal miRNA profiles may have clinical relevance for the diagnosis, prognosis, or monitoring of CRC therapy. This non-invasive liquid biopsy technique for CRC highlights the translational potential of exosomal miRNAs while underscoring the need for standardisation of the methodology.

Liquid biopsy tests currently available in the colorectal cancer (CRC) liquid biopsy field include circulating tumour DNA (ctDNA), circulating tumour cells, RNA-based markers, and protein-based assays. A recent systematic review of blood-based biomarkers for early CRC detection evaluated DNA, RNA, and protein biomarkers and highlighted both their diagnostic potential and the need for large-scale prospective validation before clinical implementation. Exosomal miRNAs, in comparison to ctDNA, may provide a view of active secretion and tumour–cell communication, rather than only tumour-cell death, as observed with ctDNA (105). This may provide a view of dynamic biological processes that may not be reflected in DNA-based liquid biopsy tests. The application of exosome miRNA liquid biopsy may provide opportunities for diagnosis, prognosis, and real-time monitoring of therapy in patients with CRC (**Figure 4**). However, ctDNA currently appears to have greater clinical utility for detecting and monitoring recurrence in CRC patients (106). Compared with circulating tumour DNA (ctDNA), exosomal miRNAs may provide insight into dynamic intercellular communication and active biological adaptation rather than predominantly reflecting tumour cell turnover or death. In contrast, ctDNA currently offers stronger evidence for molecular residual disease detection, mutation profiling, and recurrence monitoring in CRC. Circulating tumour cells provide additional information regarding viable metastatic cell populations but are technically limited by low abundance and isolation complexity. Protein-based biomarkers such as carcinoembryonic antigen remain clinically established but lack sensitivity and specificity, particularly in early-stage disease. Therefore, exosomal miRNAs should not be viewed as standalone replacements for existing liquid biopsy platforms but rather as complementary components that may provide additional biological and functional information when integrated into multimodal biomarker strategies (107).

Different liquid biopsy platforms provide distinct biological and clinical information, with varying levels of analytical maturity, scalability, and translational readiness (**Table 3**).

**Table 3:** Exosomal miRNAs with other liquid biopsy platforms in colorectal cancer (CRC).

Liquid biopsy platform	Biological information provided	Major strengths	Major limitations	Standardization/clinical readiness
Exosomal miRNAs	Reflect active intercellular communication, tumour microenvironment interactions, therapy resistance, and tumour adaptation	High biological stability due to vesicle encapsulation; detectable in multiple biofluids; may reflect dynamic tumour biology and intercellular signalling	Biological heterogeneity of EVs; lack of standardised isolation and normalisation methods; limited prospective validation; many studies retrospective or small cohort-based	Investigational; not yet established clinical biomarkers in CRC
Circulating tumour DNA (ctDNA)	Reflects tumour-derived genetic alterations and tumour cell turnover	Greater current clinical utility for recurrence detection and monitoring in CRC; established utility for molecular profiling	May primarily reflect tumour cell death rather than active biological communication processes	Currently the most clinically advanced liquid biopsy platform discussed in CRC
Circulating tumour cells (CTCs)	Reflect viable tumour cell populations in circulation	Provides information from intact tumour cells	Low abundance and technical complexity of isolation and analysis	Limited clinical implementation
Protein biomarkers (e.g., carcinoembryonic antigen)	Reflect tumour-associated protein expression	Widely used in current clinical practice	Low sensitivity and specificity, particularly for early-stage CRC	Clinically established but limited in performance
Stool-based biomarkers/stool-derived exosomal miRNAs	Reflect tumour-associated molecular alterations and occult gastrointestinal tumour shedding	Non-invasive sampling approach; potential supplementary role in CRC screening	Validation in larger cohorts remains limited; methodological variability	Investigational/supplementary role under evaluation

### 5.4 Barriers to standardisation and clinical implementation

Despite extensive investigation, few exosomal miRNA biomarkers have progressed toward clinical implementation. The major obstacle to the clinical translation of exosomal miRNAs has been the lack of standardisation in the procedures used to isolate these vesicles, which directly affects their purity and quantity (108). Additionally, there is no consensus on appropriate endogenous reference controls for normalising exosomal miRNA data, which complicates cross-study comparisons (109), biological variability further challenges clinical translation. Circulating exosomes originate from diverse cellular sources, including non-malignant tissues. It is possible that, in early-stage CRC, tumour-origin exosomes may represent only a minor fraction of circulating vesicles, potentially leading to signal dilution (110, 111). Another limitation is the continued focus on individual miRNAs rather than integrated miRNA signatures, which may oversimplify complex regulatory interactions and reduce biomarker robustness (112, 113). In terms of clinical utility, the exosomal miRNA biomarker field is currently in the early stages of development. To move this field forward, large-scale, prospective, multi-centre studies will be necessary to demonstrate the analytical validity, clinical validity, and clinical utility of exosomal miRNA biomarkers (114). Assay reproducibility, scalability, cost-effectiveness, and regulatory considerations will also be important factors for consideration (115). At present, exosomal miRNAs can be considered potential but still unproven biomarkers, meaning that their successful use in clinical applications depends on a better understanding of the mechanisms underlying exosomal miRNA biology, as well as on standardising methods for measuring them and integrating exosomal miRNAs into current molecular and clinical frameworks. Importantly, exosomal miRNAs are not currently established clinical biomarkers in colorectal cancer and remain investigational. Although preclinical and retrospective clinical studies have demonstrated promising diagnostic, prognostic, and therapeutic associations, prospective large-scale validation studies are still lacking. Therefore, their potential clinical implementation should be interpreted cautiously until analytical validity, clinical utility, and reproducibility are demonstrated in standardised prospective settings.

## 6 Methodological and analytical challenges in exosomal miRNA research

### 6.1 Exosome isolation and characterisation limitations

One major problem in the study of exosomal miRNA is the lack of consensus on the most appropriate method for isolating exosomes. The most frequently used methods include differential ultracentrifugation, size-exclusion chromatography, polymer-based precipitation, density gradient centrifugation, and immunoaffinity capture (116, 117). These procedures are significantly different in their efficiency, purity, and potential for co-precipitating contaminants with exosomes. The polymers approach is a relatively simple procedure, but its disadvantage is that proteins, lipoproteins, and ribonucleoproteins coprecipitate, and this may interfere with the study of isolated miRNA (118). Differential ultracentrifugation, although a very common approach, is a labour-intensive process and has the disadvantage that vesicle aggregation or structural changes may cause damage, affecting the content of the RNA (119). This improves vesicle purity but reduces vesicle concentration, which is often a limiting factor in the analysis of low-input clinical samples (120). Notably, differences in vesicle-isolation methods directly affect miRNA profiles, leading to poor reproducibility of findings across studies (121).

Furthermore, the biological heterogeneity of extracellular vesicles complicates the issue. Circulating vesicles include exosomes, microvesicles, and apoptotic bodies, which have overlapping sizes and molecular compositions (122). Current methods used to isolate these vesicles are not adequate to completely separate them. Thus, it is difficult to claim that specific miRNA signatures are present only in exosomes. Accordingly, several studies included in the current literature may more accurately reflect mixed extracellular vesicle populations rather than purified exosome subtypes. Throughout this review, the term “exosomal miRNAs” is therefore interpreted within the methodological limitations of the cited studies unless specific exosome characterisation criteria were reported. Recommendations, such as the “Minimal Information for Studies of Extracellular Vesicles” (MISEV), suggest using sizing, protein marker analysis, and imaging-based validation of the vesicles (123). However, these recommendations are not followed consistently, especially in retrospective studies. Furthermore, the absence of universally accepted markers for distinguishing exosomes from other extracellular vesicle subtypes further complicates interpretation of exosomal miRNA data (124). As a result, several studies refer to “exosomal miRNAs” without confirming their original vesicle type, leading to incorrect conclusions about the mechanism of action.

### 6.2 miRNA detection, quantification, and normalisation challenges

Identifying and measuring exosomal miRNAs is difficult without the right methodology (125). Most miRNA analyses use three major techniques: Next-Generation Sequencing, qRT-PCR, and Digital PCR. Each technique has its strengths and weaknesses. An advantage of using qRT-PCR to measure gene expression is its high sensitivity; however, it can introduce unintended technical biases if not properly normalised for the specific experiment. An advantage of using Next Generation Sequencing for miRNA profiling is that it enables high-throughput analysis, but low-abundance miRNAs can be missed in low-quantity clinical samples (126).

A major issue with the use of endogenous references for normalising miRNA samples from both cell culture and tissue sources remains unresolved. At the same time, there is no consensus on which endogenous reference miRNA should be used when analysing EV miRNA samples. Although exogenous spike-in controls have been validated for use across different reagent types (RNA extraction & amplification) to control for technical bias, they cannot control for differences in biological variability in EV miRNA or RNA content across different sample types. Systematic bias in normalising miRNA normalisation may lead to increased false-positive results (127).

### 6.3 Pre-analytical variability and data interpretation pitfalls

The pre-analytical variables have been found to affect the exosomal miRNA analysis significantly. The blood collection tubes, time, centrifugation, storage conditions, etc., have been found to affect the concentration of exosomes in the blood sample (128). Minor variations in pre-analytical conditions have been found to result in significant variations in miRNA profiles. Another source of variability is found at the analytical phase. Comparisons of computational tools used in miRNA sequencing data analysis have found discrepancies in the results. Lack of proper reporting of the analytical workflow and the use of non-standardised pipelines have been found to hinder comparisons (129, 130).

Beyond technical considerations, interpreting exosomal miRNA data poses conceptual challenges. Detection of a miRNA in exosomal preparations does not necessarily imply functional delivery or regulatory activity in recipient cells. Quantitative analyses suggest that many EVs contain few or no copies of specific miRNAs, raising questions about biological sufficiency (131). Furthermore, many of these studies tend to focus on correlative associations rather than mechanistic relevance, and these approaches are prone to biological overinterpretation, in which passive molecular association is mistaken for actual biological signalling (132). There is an increasing need for translational studies that rigorously evaluate dose, functional impact, and *in vivo* relevance of proposed exosomal miRNA mechanisms.

Collectively, these methodological and conceptual limitations continue to hinder robust clinical translation of exosomal miRNA research. Improved standardisation, quality control, and functional validation will be essential for establishing clinically reliable exosomal miRNA biomarkers (133). Until these barriers are overcome, results should be viewed with caution, especially when clinical relevance is claimed. The principal methodological variables influencing exosomal miRNA reproducibility and translational robustness are summarised in **Table 4**.

**Table 4:** Methodological and technological challenges affecting reproducibility and clinical translation of exosomal miRNA research in CRC.

Methodological parameter	Source of variability	Impact on exosomal miRNA analysis	References
Exosome isolation method (ultracentrifugation, density gradient, size-exclusion chromatography, precipitation, immunoaffinity capture)	Differences in yield, purity, and contaminant co-isolation	Alters vesicle composition and detected miRNA profiles; limits cross-study reproducibility	(116–121)
Co-isolation of non-exosomal particles	Lipoproteins, protein aggregates, ribonucleoproteins	Potential misattribution of miRNAs to exosomes	(119, 120)
Incomplete separation of EV subtypes	Overlap between exosomes, microvesicles, and apoptotic bodies	Ambiguity in assigning functional relevance	(121–124)
Inconsistent adherence to MISEV guidelines	Variable vesicle characterisation standards	Reduced comparability across studies	(123)
miRNA detection platforms (qRT-PCR, NGS, digital PCR)	Platform-specific sensitivity and bias	Variability in miRNA quantification	(125, 126)
Lack of consensus on endogenous controls	No stable reference miRNA for normalisation	Systematic bias; false-positive findings	(126, 127)
Pre-analytical variables (blood tube type, processing time, storage conditions)	Sample handling differences	Significant alterations in miRNA yield and profile	(128)
Bioinformatic pipeline variability	Differences in sequencing data processing	Inconsistent abundance estimates and differential expression results	(129, 130)
Functional overinterpretation of detected miRNAs	Low copy number per vesicle; correlative analyses	Risk of overstating biological relevance	(131, 132)
Limited standardisation and validation	Heterogeneous protocols and small cohorts	Barriers to clinical translation	(133)

## 7 Therapeutic targeting of exosomal miRNA pathways in CRC

### 7.1 Exosome-based delivery of tumour-suppressive miRNAs

The therapeutic application of exosomal miRNAs as functional regulators of intercellular communication has been of significant interest in the field of miRNA therapy. The application of miRNA therapy in the form of exosomes has the advantage of regulating gene expression post-transcriptionally, thus influencing multiple downstream pathways simultaneously (134). Exosomes possess several characteristics that make them attractive delivery vehicles, including biocompatibility, low immunogenicity, endogenous RNA-carrying capacity, and the ability to transfer biologically active RNA molecules to recipient cells, and can also be further engineered for targeted delivery applications (135). Experimental studies have shown that exosomes can transfer miRNAs that repress target gene expression and modulate tumour cell phenotype *in vivo*. This suggests that exosomes have great potential as RNA-based therapies (136, 137). Despite this promising potential, the therapeutic application of exosomal miRNA remains in its infancy, with many biological hurdles to be overcome.

As therapy with nucleic acids improves, exosomes are becoming a promising vehicle for therapy. Exosomes have been found to have potential advantages over synthetic nanoparticles, including reduced immunogenicity, a shorter blood half-life, and a lack of tissue specificity (138, 139). However, it still has to be seen if exosomes can offer any advantages over existing vehicles by a direct comparison. Preclinical models have explored the use of exosomes to deliver tumour-suppressive miRNAs that are downregulated in CRC. Exosomes loaded with specific miRNAs can inhibit tumour growth, invasion, and metastatic progression by suppressing oncogenic signalling pathways in CRC cells (140).

Various approaches have been investigated for loading miRNAs into exosomes, including electroporation, direct transfection, and donor cell engineering (141). Among them, genetic modification of donor cells ensures more stable packaging of miRNAs in exosomes and minimises vesicle damage compared with post-isolation loading techniques (142). However, the efficiency of loading, as well as its reproducibility and scalability, varies among different platforms. Another issue that needs to be addressed is the control of dosage and pharmacokinetics. Unlike conventional therapeutic molecules, the concentration of miRNAs delivered to cells via exosomes is difficult to determine, and their pharmacokinetic parameters are not well defined (143). This uncertainty can create complexities in optimisation, safety assessment, and regulation.

### 7.2 Inhibition of oncogenic exosomal miRNAs and secretion pathways

Another therapeutic strategy is to target oncogenic exosomal miRNAs by inhibiting their production, release, or activity (144). In CRC models, antisense oligonucleotides and miRNA sponges targeting specific miRNAs reduce tumour growth and enhance chemosensitivity, supporting the feasibility of targeting exosomal miRNA signalling (145). Another way to reduce/limit exosome production and release from cells has been through interventions at the levels of biogenesis and secretion. Experimental models have shown that both neutral sphingolipid inhibition and disruption of ESCRT-related proteins decrease exosome release and diminish tumour progression (146). Given that these pathways are important in other vital physiological processes, researchers must be cautious about the potential for systemic toxicity during the process, especially when the therapeutic approach considered is the inhibition or reduction of exosome secretion.

Inhibition of exosome secretion is unlikely to be a viable clinical strategy, as EVs play critical roles in immune regulation, tissue homeostasis, and normal intercellular communication (147). Therefore, selective therapeutic strategies that preferentially interfere with exosomal signalling arising from tumour cells while preserving the normal function of EVs pose a significant challenge for clinicians.

### 7.3 Engineering, safety, and translational barriers

Significant progress has also been made in bioengineering exosome surfaces for improved tissue specificity and uptake efficiency. Proof-of-concept studies have shown that exosomes modified to enhance miRNA uptake into cancer cells are efficient at reducing off-target miRNA distribution (148, 149). For CRC, approaches have been developed for exosome-based miRNA delivery that exploit molecules enriched on tumour cells and tumour-associated vasculature. However, much remains to be understood about exosome manufacturing and quality control before such approaches are fully explored. The long-term safety and immunogenicity of exosomes have not been fully explored (150, 151).

Scalability represents a major translational bottleneck. Current exosome engineering platforms are labour-intensive and difficult to standardise for large-scale production, limiting their readiness for clinical deployment (152). Regulatory pathways for extracellular vesicle-based therapeutics are still in development, and approval pathways for biologically complex therapeutics are not well defined (153). Considering these existing limitations, it would be pertinent to discuss exosomal miRNA therapy in terms of long-term goals rather than short-term objectives. Although biological activity has been confirmed, there are still concerns regarding biodistribution, dosing, pharmacodynamics, and safety profiles (154–156). At this point, exosomal miRNA therapeutics should be considered adjunctive therapies that might complement existing treatments rather than replace them (157). To translate these therapeutics into effective therapeutic strategy, it is necessary to standardise their production, compare them with existing RNA therapeutics, and validate them. Until these challenges are addressed, therapeutic strategies targeting exosomal miRNAs in CRC should be considered with caution. A list of current therapeutic strategies targeting exosomal miRNA pathways and their translational limitations is summarised in **Table 5**.

**Table 5:** Experimental therapeutic strategies targeting exosomal miRNA pathways in CRC and their translational limitations.

Therapeutic strategy	Target level	Mechanism of action	Experimental evidence described	Key limitations	References
Exosome-mediated delivery of tumour-suppressive miRNAs	miRNA replacement	Restoration of downregulated tumour-suppressive miRNAs via engineered or donor-cell–derived exosomes	Suppression of tumour growth and invasion in preclinical models	Loading efficiency, dose control, and pharmacokinetics are unclear	(140–143)
Antisense oligonucleotides/miRNA sponges	Oncogenic exosomal miRNAs	Inhibition of specific miRNAs associated with tumour progression	Reduced tumour growth and increased chemosensitivity in CRC models	Delivery specificity; off-target effects	(144, 145)
Inhibition of exosome biogenesis/secretion	Vesicle production pathways	Targeting neutral sphingomyelinase or ESCRT-related proteins to reduce exosome release	Attenuation of tumour progression in experimental systems	Interference with physiological vesicle functions	(146, 147)
Engineered exosomes with targeting ligands	Targeted delivery systems	Surface modification to enhance tumour-specific uptake	Improved delivery efficiency in preclinical models	Scalability; safety; immunogenic considerations	(148) (151),
Regulatory and manufacturing considerations	Production and quality control	Standardisation of exosome preparation and potency assays	Identified as critical translational barriers	Batch variability; undefined regulatory pathways	(152–157)

## 8 Future perspectives: systems biology, precision oncology, and translational design principles

### 8.1 Network-level and multi-omics integration

Another recurring drawback of existing exosomal miRNA-based research is that it has mostly focused on individual miRNAs as independent regulators of gene expression. However, it is known that miRNAs function as part of highly connected and complex gene regulatory systems, wherein each miRNA targets multiple genes and does not predominantly regulate a single gene of interest. Thus, studies focusing on individual miRNAs might not fully elucidate the complexity of tumour adaptations. In CRC, recent studies employing systems biology approaches that integrate exosomal miRNA profiles with transcriptome and proteome profiles have identified tumour progression and therapy resistance-related miRNA-based regulatory modules (158). This would imply that miRNA-based signatures could be more robust and information-rich than miRNA-based markers. In a more practical sense, systems biology approaches could enable more reliable biomarkers by reducing biological and technical variability. Multivariate models incorporating miRNA networks may also better reflect dynamic tumour behaviour under therapeutic pressure (159).

### 8.2 Resolving exosome heterogeneity and spatial-temporal dynamics

One of the major issues to be addressed in relation to EVs is that they are heterogeneous in nature. It has been found that exosomes are heterogeneous in nature and comprise different subtypes that have different pathways in biogenesis and functional potential (160). Bulk isolation of EVs may mask the heterogeneity of EVs and may ignore active subtypes. Recent advances in single-vesicle analysis, high-resolution flow cytometry, and super-resolution imaging provide new opportunities to dissect exosome heterogeneity with greater precision (161). Applying these technologies to CRC-derived vesicles may help determine which exosome subpopulations carry functionally relevant miRNAs and which represent background or non-functional vesicle populations.

Another aspect affecting exosomal miRNA signalling is spatial and temporal dynamics. Most studies have relied on static analyses at a single time point, whereas tumour progression and therapy adaptation are dynamic processes. Longitudinal analysis of exosomal miRNAs before, during, and after treatment can identify potential temporal signatures of response, adaptation, or relapse (162). Spatial context is equally important. The biological activity of exosomal miRNAs may vary depending on whether communication is within the primary tumour microenvironment, in circulation, or in distant metastatic microenvironments (163). Elucidation of how spatial transcriptomics, imaging-based techniques, and exosomal miRNAs intersect could provide insights into where exosomal miRNAs have a functionally relevant activity (164). Resolving these spatial and temporal aspects is crucial for enhancing translational accuracy. For clinical purposes, selective targeting or detection of tumour-derived vesicle subsets may be more potent than studying the total vesicle population (165).

### 8.3 Artificial intelligence, precision oncology integration, and design principles

The complexity and high dimensionality of exosomal miRNA datasets necessitate advanced analytical methodologies. Machine learning and artificial intelligence (AI) approaches are increasingly employed to identify multivariate biomarker signatures that outperform traditional univariate analyses (166, 167).

In CRC, early AI-based models applied to circulating miRNA datasets have demonstrated improved diagnostic and prognostic performance compared with conventional statistical approaches (168). However, overfitting is one major issue to be concerned about, especially in research with low sample sizes. For translational application, it has been noted that in the future, design and validation cohorts should be emphasised in any computational model (169). Finally, exosomal miRNAs should be considered in precision oncology rather than in a single-biomarker fashion. The application of exosomal miRNA profiling in conjunction with other tools, such as circulating DNA, imaging, and clinicopathological features, could help improve risk stratification and evaluate treatment efficacy (170). Future translational studies should also consider the molecular and clinical heterogeneity of CRC. Distinct molecular subtypes, including microsatellite instability-high (MSI-H) and microsatellite stable (MSS) tumours, as well as consensus molecular subtype (CMS) classifications, exhibit differences in immune activity, stromal composition, metastatic behaviour, and therapeutic responsiveness. These subtype-specific biological differences may influence exosome secretion patterns, exosomal miRNA composition, and tumour microenvironment interactions. Similarly, the clinical relevance of exosomal miRNAs may vary across disease settings, including early-stage disease, metastatic CRC, minimal residual disease monitoring, and treatment response assessment. Incorporation of molecular subtype and clinical context into future study design may improve the biological interpretation and translational relevance of exosomal miRNA-based biomarkers. In light of the current evidence base, the following principles should shape future research in the field: 1. Mechanism first validation: The correlative evidence should be subjected to functional as well as *in vivo* validation. 2. Standardisation and transparency: Standardised methods should be used for the handling of the sample, isolation of the vesicles, and the analysis. 3. Network-based interpretation: Instead of the use of the miR-based methods, systems-based methods should be used. 4. Longitudinal and spatial design: The studies should include the temporal as well as the spatial aspects. 5. Clinical relevance: The endpoints should include the therapeutic response as well as the recurrence.

Adherence to these principles may facilitate the transition of exosomal miRNA research from exploratory discovery toward clinically actionable applications. The key emerging concepts and translational design principles proposed to strengthen exosomal miRNA research are summarised in **Table 6**.

**Table 6:** Emerging concepts and translational design principles for improving exosomal miRNA research in CRC.

Emerging concept	Current limitation addressed	Proposed advancement	Supporting reference(s)
Network-level miRNA analysis	Overreliance on single-miRNA models	Systems biology and multi-omics integration	(158, 159)
Resolution of exosome heterogeneity	Bulk vesicle analysis obscures functional subsets	Single-vesicle technologies and high-resolution profiling	(160, 161)
Spatial–Temporal Profiling	Static single-timepoint measurements	Longitudinal sampling and spatial transcriptomics integration	(162–164)
AI-driven biomarker discovery	High-dimensional data complexity	Machine learning models with independent validation	(166–169)
Integration into precision oncology	Isolated biomarker interpretation	Combination with ctDNA, imaging, and clinicopathological data	(170)

## 9 Conclusion

Exosomal miRNAs have emerged as key regulators of CRC biology and are recognised as active mediators of intercellular communication rather than passive molecular byproducts. The evidence suggests that tumour-derived exosomal miRNAs modulate multiple hallmarks of cancer, including EMT, metastatic niche formation, immune responses, metabolic remodelling, and chemo-radio-resistance. Through horizontal transfer of regulatory signals, these vesicles enable coordinated adaptation among tumour cell populations and dynamic reshaping of the tumour microenvironment.

Significantly, exosomal miRNAs also contribute to the maintenance of stemness and plasticity in CRC cells. This is because exosomal miRNAs reinforce self-renewal and stress-adaptive pathways, thereby ensuring tumour heterogeneity and long-term therapeutic resistance. This review proposes a population-level framework to tumour evolution, in which exosomal communication is crucial for the survival of tumour populations under selective and therapeutic pressures. Despite considerable biological and translational interest, clinical implementation of exosomal miRNAs remains constrained by methodological variability, extracellular vesicle heterogeneity, lack of standardised normalisation strategies, and insufficient mechanistic validation. Also, the focus on individual miRNAs has oversimplified complex regulatory networks, thereby compromising biomarker stability.

Addressing these challenges requires a shift toward mechanism-driven and integrative research strategies. The incorporation of multi-omics approaches, single-vesicle analyses, spatial-temporal profiling, and artificial intelligence-based modelling, alongside standardised protocols and validation in diverse cohorts, would be essential. Within the framework of precision oncology, exosomal miRNAs should be viewed not as standalone solutions, but as components of a broader diagnostic and therapeutic toolkit.

In summary, exosomal miRNAs represent a promising and emerging area of CRC research that deserves focused attention. When studied using robust experimental designs and rigorous methodological approaches, they hold significant potential for the development of novel biomarkers and therapeutic strategies. However, realising this potential requires careful consideration of the biological and methodological complexities inherent to CRC research.
